# The in situ near-total pancreatectomy (LIVOCADO procedure) for end-staged chronic pancreatitis

**DOI:** 10.1007/s00423-021-02107-x

**Published:** 2021-06-25

**Authors:** Ryan D. Baron, Andrea R. G. Sheel, Ammad Farooq, Jörg Kleeff, Pietro Contin, Christopher M. Halloran, John P. Neoptolemos

**Affiliations:** 1 Department of Pancreato-Biliary Surgery, The Royal Liverpool University Hospital, Liverpool, UK; 2 Department of Clinical Cancer Medicine, Institute of Translational Medicine, The University of Liverpool, Liverpool, UK; 3 Department of Radiology, The Royal Liverpool University Hospital, Liverpool, UK; 4 Department of Visceral, Vascular and Endocrine Surgery, Martin, Halle (Saale), Germany; 5 Department of General, Visceral and Transplantation Surgery, University of Heidelberg, Im Neuenheimer Feld 420, 69120 Heidelberg, Baden-Württemberg, Germany

**Keywords:** Surgery, Extra-hepatic portal hypertension, Pain, Varices, Beger operation, Frey operation

## Abstract

**Purpose:**

Total pancreatectomy for severe pain in end-stage chronic pancreatitis may be the only option, but with vascular involvement, this is usually too high risk and/or technically not feasible. The purpose of the study was to present the clinical outcomes of a novel procedure in severe chronic pancreatitis complicated by uncontrollable pain and vascular involvement.

**Methods:**

We describe an in situ near-total pancreatectomy that avoids peripancreatic vascular dissection (Livocado procedure) and report on surgical and clinical outcomes.

**Results:**

The Livocado procedure was carried out on 18 (3.9%) of 465 patients undergoing surgery for chronic pancreatitis. There were 13 men and 5 women with a median (IQR) age of 48.5 (42.4–57) years and weight of 60.7 (58.0–75.0) kg. All had severe pain and vascular involvement; 17 had pancreatic parenchymal calcification; the median (IQR) oral morphine equivalent dose requirement was 86 (33–195) mg/day. The median (IQR) maximal pain scores were 9 (9–10); the average pain score was 6 (IQR 4–7). There was no peri-operative or 90-day mortality. At a median (IQR) follow-up of 32.5 (21–45.75) months, both maximal and average pain scores were significantly improved post-operatively, and at 12 months, two-thirds of patients were completely pain free. Six (33%) patients had employment pre-operatively versus 13 (72%) post-operatively (*p* = 0.01).

**Conclusions:**

The Livocado procedure was safe and carried out successfully in patients with chronic pancreatitis with vascular involvement where other procedures would be contraindicated. Perioperative outcomes, post-operative pain scores, and employment rehabilitation were comparable with other procedures carried out in patients without vascular involvement.

## Introduction

Chronic pancreatitis is a complex inflammatory syndrome of the pancreas with pain as the predominant symptom [1]. It affects individuals with genetic, environmental, and/or other risk factors who develop persistent pathological responses to parenchymal injury or stress [2, 3]. CP is a major source of morbidity with the incidence and prevalence estimated to be around 5–12 per 10^5^ per year and 50 per 10^5^, respectively [4–7]. Chronic pancreatitis carries a heavy disease burden including chronic pain; pancreatic endocrine and exocrine failure leading to diabetes mellitus and malnutrition; lower quality of life; serious long-term complications including a 5–25-fold risk of pancreatic cancer; and social stigma, with a reduced life expectancy [8–10].

The long-term morphological sequelae of chronic inflammation, fibrosis, and loss of parenchymal architecture result in ductal and parenchymal calcifications, ductal strictures, inflammatory masses, pseudocysts, biliary and duodenal obstruction, pancreatic fistulae, and pancreatic ascites [2, 3, 8, 9]. Vascular complications include porto-mesenteric venous compression or occlusion, extra-hepatic portal hypertension, splenic-portal-thrombosis, venous collateralization, and pseudoaneurysm [2, 11–14]. Longitudinal studies show that 40–75% of CP patients require surgical intervention most commonly for intractable pain [15–18]. Duodenum- preserving pancreatic head resection (DPPHR) notably the Beger, Frey, and Berne procedures are effective for head-dominant disease, providing decompression of the duodenum, hepatic portal vein, main pancreatic duct, and intra-pancreatic bile duct [19–22].

There remains a role for total pancreatectomy in a highly select group of patients with end-staged CP affecting the entire pancreas, intractable pain, and preexisting endocrine failure [2, 23–25]. Total pancreatectomy in chronic pancreatitis is however associated with substantial morbidity and mortality especially in cases with vascular involvement. The aim of this study was to evaluate the early and late outcomes of a novel surgical procedure which offers an alternative for patients who would otherwise require a total pancreatectomy. This procedure combines a duodenum-preserving pancreatic head resection with extended coring of the neck, body, and tail of the pancreas, leaving only an outer rim of fibrosed tissue, which is anastomosed to a Roux-en-Y jejunal limb.

## Methods

### Study design

This is a single-centre cohort series of consecutive patients with chronic pancreatitis referred to the Liverpool Pancreas Centre for further evaluation between January 1997 and May 2020.

The in situ near-total pancreatectomy procedure was first introduced on 30 December 2014, and the last procedure was undertaken on 11 February 2020. The data lock for all patients was on 18 May 2020. A prospectively maintained database recorded demographic, clinical, radiological, genetic, and histopathological data along with the patient’s performance status and employment status during initial patient clinical assessment in the pancreas outpatient clinic. All patients were asked to complete patient-reported pain scores on visual analogue scale scores recorded on a 10-point Likert scale (0–10), including maximal (“worst”) pain and average pain. Patients were followed up after discharge in accordance with local clinical protocol, which comprised of routine assessment at 4–6 weeks, 3, 6, and 12 months, then annually with additional review as clinically required. Data collected at follow-up included weight, diabetes status, presence of steatorrhea, pancreatic enzyme replacement dosage, analgesia requirements, employment status, and pain scores. The equianalgesic equivalence to oral morphine was calculated for all opiate medications as recommended by the Royal College of Anaesthetists of England [26]. Complications were graded according to Dindo et al. [27]. Data were censored at the point when patients were discharged, lost to follow-up, or died.

### Diagnosis of chronic pancreatitis

The diagnosis of CP was based on clinical and radiological criteria and confirmed in all patients following histopathological assessment of operative specimens [28, 29]. CP secondary to alcohol required alcohol consumption  ≥ 62 units per week for  ≥ 1 year [30]. Patients with idiopathic CP were classified into two groups: (1) idiopathic with no genetic background and with a genetic background [31]. The presence of pancreatic exocrine insufficiency was based on clinical assessment, and the response of steatorrhea to pancreatic enzyme replacement therapy.

### Radiological review

Vascular assessment was made on the basis of a pre-operative pancreas protocol CT including arterial and portal venous phase imaging. All CT scans were reviewed and scored retrospectively by a specialist pancreatic radiologist blinded to patient outcomes [28].

### Eligibility criteria

The in situ near-total pancreatectomy procedure was only considered in a highly selected small subset of patients that had severe end-stage chronic pancreatitis where the whole pancreas was diseased with exocrine and endocrine insufficiency and daily debilitating abdominal pain unresponsive to medical treatment, and abstinence from alcohol for more than 6 months.

### Operative description

**Fig. 1:**
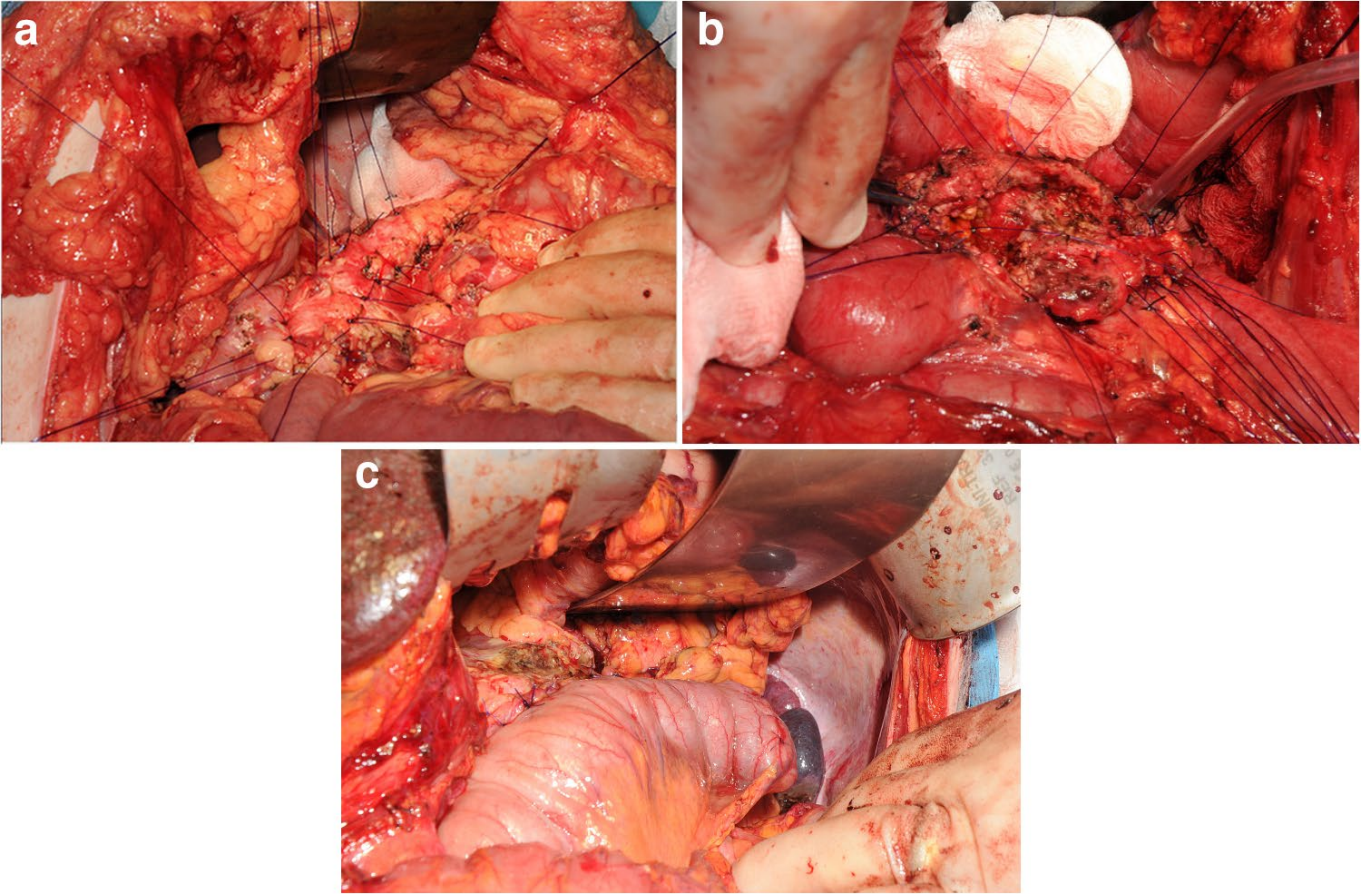
Operative photographs demonstrating the key stages of the Livocado procedure. **a** The ventral pancreas is exposed and hemostatic sutures are placed around the entire pancreatic margin. **b** A duodenum-preserving pancreatic head resection with near-total coring extended across the pancreatic neck and along the length of the body and tail is performed. **c** Longitudinal pancreato-jejunostomy using a Roux-en-Y reconstructive limb

The duodenum and pancreas were exposed as described previously and the duodenum was fully Kocherized [23]. The pancreatic margins were defined by dividing the superior and inferior peritoneal reflections and the right gastroepiploic vein (or the gastrocolic trunk of Henle when required) was ligated and divided to fully reflect the antrum of the stomach off the anterior head of the pancreas. Hemostatic sutures are placed around the entire pancreatic margin (Fig. 1a). The pancreatic head was cored out following the principles of the Berne modification of the Beger procedure [32]. This resection was continued across the neck of the pancreas taking extreme care of the superior mesenteric, hepatic portal venous axis, and along the entire length of the body and tail of the pancreas. All pancreatic tissue anterior to the main pancreatic duct and as much of the tissue superior, inferior, and posterior to the duct as possible was cored out leaving only a thin fibrotic outer rim of pancreas. Because of the dense fibrous tissue and calcification, a combination of sharp dissection with a scalpel and scissors was required. This is analogous to a cored-out avocado providing the term Livocado in part reference to its origin in Liverpool. A cholecystectomy was then performed, and the cystic duct was catheterized using an umbilical feeding catheter. This tube was palpated within the cored-out pancreatic head and the intra-pancreatic bile duct was incised and widely marsupialized using 4 to 6 interrupted 4–0 sutures (Fig. 1b). The jejunum was divided using a linear cutter-stapler, and the distal limb was delivered through an incised transverse mesocolon defect as a Roux-en-Y. The jejunal limb was opened by a diathermy longitudinal incision along the anti-mesenteric border and sutured to the pancreatic rim using continuous 4–0 PDS sutures between stays as follows. The distal end of the limb was first parachuted to the tip of the tail of the pancreas using interrupted stay sutures. The inferior pancreatic rim was then sutured to the jejunal enterotomy using a continuous suture and full thickness bites, across the neck and around the inferior aspect of the cored-out head and uncinated process. The superior border of the pancreatic rim was then continuously sutured to the jejunal enterotomy again from tail to head. Along the pancreatoduodenal groove, the jejunal enterotomy could be sutured to the medial duodenal wall if needed. The superior and inferior sutures were then tied together when meeting (Fig. 1c). The gastroduodenal limb was then anastomosed side-to-side to the pancreatic limb to complete the Roux-en-Y.

### Eligibility criteria

The in situ near-total pancreatectomy procedure was only considered in a highly selected small subset of patients that had all of the following criteria.

1. severe end-stage chronic pancreatitis where the whole pancreas was diseased with exocrine/endocrine failure;
2. daily debilitating abdominal pain unresponsive to medical treatment;
3. duodenum- and spleen-preserving near-total pancreatectomy or standard total pancreatectomy was technically not feasible, notably due to vascular and/or other intra-abdominal complications;
4. demonstrable abstinence from alcohol for more than 6 months.

### Statistical analysis

Continuous variables are presented as median and interquartile range (IQR). Statistical comparison was undertaken using the Wilcoxon rank test for paired data based on a 2-tailed alpha. Categorical variables are presented as frequency and percentage and were analyzed using the *Χ*^2^ test, or Fishers exact probability test. Significance was set at the 5% level (*p* < 0.05). SPSS v24 was used for the analyses.

## Results

### Patient demographics and chronic pancreatitis characteristics

Between January 1997 and May 2020, approximately 1200 patients with a diagnosis of chronic pancreatitis were referred to the Liverpool Pancreas Centre for further evaluation. Surgery was undertaken in 465 patients comprising a pylorus preserving partial pancreato-duodenectomy in 133 (28.6%), a Beger’s duodenum head resection in 130 (28.0%), a classical Kausch-Whipple pancreato-duodenectomy in 8 (1.7%) patients, a left pancreatectomy (with or without spleen preservation) in 43 (9.3%), various drainage procedures in 67 (14.4%) patients (including Partington-Rochelle, Izbicki V-procedure, and pseudocyst-jejunostomy), and total pancreatectomy in 66 (14.2%) patients (including duodenum- and spleen-preserving near-total pancreatectomy in 51). The remaining 18 (3.9%) patients (13 men and 5 women) underwent a Livocado resection all with severe uncontrollable pain as the primary indication, with baseline demographic and operative details shown in Table 1. The median (IQR) age was 48.5 (42.4–57) years and median (IQR) weight of 60.7 (58.0–75.0) kg and a BMI of 21.2 (20.1–25.5). The median (IQR) duration of symptoms at the time of surgery was 4 (2–10.3) years. The aetiology was excess alcohol in 12 with a prior median (IQR) consumption of 200 (100–245) units per week. Six patients were idiopathic of whom one had a genetic background (a heterozygous SPINK-1 pAsn34Ser variant and a heterozygous CFTR pArg117His mutation). Seventeen patients had a history of tobacco smoking, of whom 13 were current smokers, with a median (IQR) of 26.3 (19.2–37) pack years; one patient had never smoked.

**Table 1:** Details of patient baseline demographic, operative details, and outcomes

Clinical variables	Frequency
Total patients	18
Men	13 (72%)
Age, years: median (IQR)	48.5 (42.5–57.0)
Weight, kg: median (IQR)	60.7 (58.0–75.0)
Body mass index: median (IQR)	23.8 (21.3–27.8)
Symptoms	
Primary symptom severe pain	18 (100%)
Pancreatic exocrine insufficiency	18 (100%)
^1^PERT, lipase units: median (IQR)	290,000 (225,000–350,000)
Diabetes mellitus	11 (61%)
Risk factors	
Alcohol (> 62 units per week for > 1 year): median (IQR)	12 (67%)
Alcohol, units/week: median (IQR)	200 (100–245)
Idiopathic/risk mutation	6 (33%)
Current smokers	13 (72%)
Ever smokers	17 (94%)
Pack years: median (IQR)	26.3 (19.2–37.0)
Previous surgery	
Beger’s procedure	2 (11%)
Splenectomy	1 (6%)
Minimal access retroperitoneal necrosectomy	1 (6%)
EUS-guided pseudocyst-duodenostomy	1 (6%)
Analgesia: equianalgesic morphine dose, mg/day: median (IQR)	86 (33–195)
Pre-operative pain score	
Maximal pain: median (IQR)	9 (9–10)
Average pain: median (IQR)	6 (4–7)
Performance status	
0	7 (39%)
1	5 (28%)
2	4 (17%)
3	2 (11%)
4	1 (6%)
ASA grade	
I	1 (6%)
II	13 (72%)
III	4 (22%)
Pre-op employment status: employed	6 (33%)
Radiological imaging	
Vascular involvement	18 (100%)
Porto-mesenteric vein occlusion	2 (11%)
Porto-mesenteric vein compression	10 (56%)
Splenic vein occlusion	5 (28%)
Splenic vein compression	12 (67%)
Extrahepatic portal hypertension	13 (72%)
Portal and/or gastrosplenic varices	17 (94%)
Portal varices and/or cavernous transformation	12 (67%)
Gastrosplenic varices	16 (89%)
Splenomegaly	9 (50%)
Arterial involvement	1 (6%)
Ascites	4 (22%)
Pancreatic atrophy	18 (100%)
• Mild (< 50%) atrophy	7 (39%)
• Moderate (50–75%) atrophy	6 (33%)
• Severe (> 80%) atrophy	5 (28%)
• Pancreatic atrophy, %	60 (22.5–70)
Pancreatic calcification	17 (94%)
• Head	17 (94%)
• Neck	16 (88%)
• Body	14 (78%)
• Tail	13 (72%)
Pancreatic duct dilatation/stricture	12 (67%)
Fluid collection	11 (61%)
Pseudocysts	10 (56%)
Inflammatory head mass	8 (44%)
Biliary obstruction	5 (28%)
Gastric outlet obstruction	5 (28%)
Pancreato-peritoneal fistula	1 (6%)
Operative details	
Operation duration: median (IQR)	6 h 37 m (5 h 17 min–7 h 10 min)
Overall blood transfusion, units: median (IQR)	0 (0–3)
Patients blood transfused	8 (44%)
Median (IQR) blood transfusion in the 8 transfused	3 (1.25–5.75)
Splenectomy performed	4 (22%)
Post-operative complications (Clavien-Dindo)	
Any complication	9 (50%)
I	2 (11%)
II	5 (28%)
IIIa/b	1 (6%)
IVa/b	1 (6%)
V	0 (0%)
Hospital stay: days, median (IQR)	13.5 (10–21.3)
Follow-up	
Length of follow-up, months: median (IQR)	32.5 (21–45.8)
Diabetes mellitus	17 (94%)^2^
Pancreatic exocrine insufficiency	18 (100%)
PERT, lipase units^1^	325,000 (242,500–450,000)^3^
Post-op employment status: employed	13 (67%)^4^

^1^PERT = pancreas enzyme replacement therapy

^2^Significant compared to pre-operative status *p* = 0.04

^3^Significant compared to pre-operative status* p* = 0.015

^4^Significant compared to pre-operative status *p* = 0.01

All 18 patients suffered with severe pain with a median (IQR) oral morphine equivalent dose of 86 (33–195) mg/day. The median (IQR) patient-reported pain scores were 9 (9–10) for the maximal pain score and 6 (IQR 4–7) for the average pain score. All 18 patients had pancreatic exocrine insufficiency requiring pancreatic enzyme replacement therapy with a median (IQR) dose of 290,000 (225,000–360,000) lipase units per day. Eleven patients (61%) had overt pre-operative diabetes mellitus, eight requiring subcutaneous insulin and three required oral anti-hyperglycemic medication and seven had pre-diabetes. Twelve patients had a median (IQR) weight loss of 7.5 (5–9.5) kg. Five patients had radiological biliary obstruction, and two were clinically jaundiced.

### Pre-operative radiological findings

**Fig. 2:**
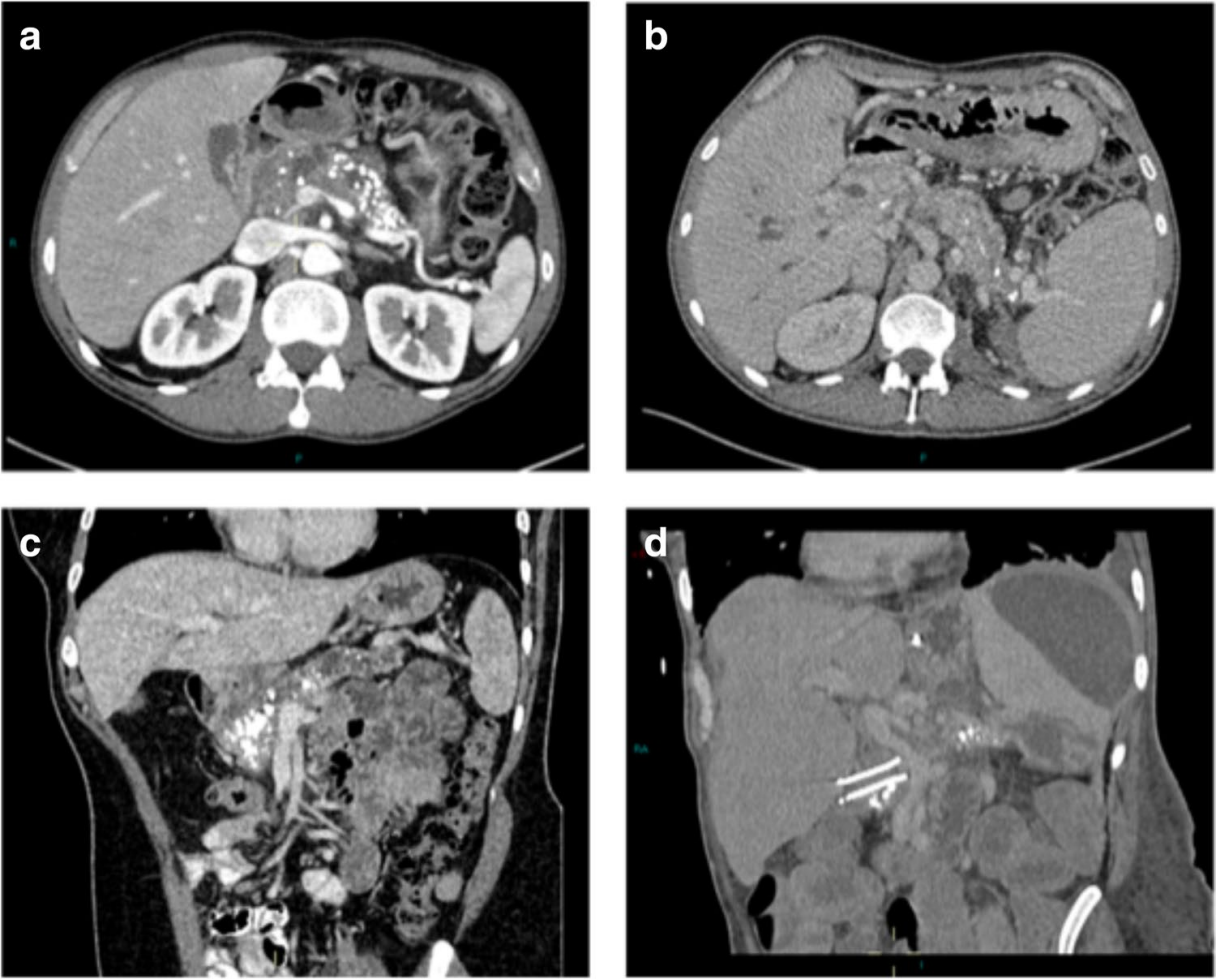
Pre-operative CT images from four different patients. **a** Parenchymal atrophy and main pancreatic duct dilatation with diffuse parenchymal and ductal calculi. Stenosis of the splenic vein and varices. **b** Hepatic portal and splenic vein thrombosis, and splenomegaly, with splenic and gastric vein varices. Extra-hepatic bile duct occlusion with intra-hepatic duct dilatation and a previous left nephrectomy. **c** Pancreatic parenchymal atrophy with diffuse pancreatic parenchymal and ductal calculi and upstream main pancreatic duct dilatation. Splenic and gastric vein varices. **d** Duodeno-pseudocystic covered stent, non-occlusive hepatic vein thrombus and splenic vein occlusion, upper abdominal varices, splenomegaly with inferior pole infarction and large subcapsular collection. Left-sided pleural effusion

All 18 patients had end-stage chronic pancreatitis with vascular involvement (representative images from selected patients are presented in Fig. 2). Seventeen (94%) patients had varices, hepatic portal varices/cavernous transformation in 12 (67%), and gastrosplenic varices in 16 (89%); 11 patients had both hepatic portal and gastrosplenic varices. Twelve (66%) patients had venous stenosis or occlusion, affecting the splenic vein in all 12 patients and the hepatic portal vein/superior mesenteric vein axis in 10 cases. Two (11%) patients had complete portal vein occlusion and 5 (28%) patients splenic vein occlusion. Nine (50%) patients had splenomegaly and four (22%) patients had ascites. One patient had arterial involvement with significant inflammation around the superior mesenteric artery.

All patients demonstrated pancreatic atrophy estimated radiologically as mild (< 50%) in 7 (39%) patients, moderate (50–75%) in 6 (33%) patients, and severe (> 75%) in 5 (28%) patients. The median (IQR) radiologically estimated atrophy was 60% (22.5–70%).

Seventeen (94%) patients had pancreatic parenchymal calcification affecting the head in all 17 (94%), the neck in 16 (89%), the body in 14 (78%), and the tail in 13 (72%). Ten (56%) patients had main pancreatic duct dilatation which affected the pancreatic neck in all 10 patients, the body in 8 patients, the head in 4 patients, and the tail in 5 patients. Two patients had main pancreatic duct strictures, both in the pancreatic neck.

Eleven (61%) patients had peripancreatic fluid collections, 10 (56%) had pseudocysts, and 8 (44%) had an inflammatory mass of the pancreas.

Seven (39%) patients had peripheral organ involvement, 5 (28%) with biliary obstruction, 5 (28%) with radiologic gastric outlet obstruction, and one patient had an internal pancreato-peritoneal fistula.

### Patient fitness, previous intervention, and operative outcomes

Twelve (67%) patients had a performance status of 0 or 1, three patients had a performance status of 2, two had a performance status of 3, and one patient had a performance status of 4. The ASA grade for 14 patients (77.8%) was 1 or 2 and four patients were ASA grade 3.

Four patients had undergone previous pancreatic intervention including a Berne modification procedure in two patients, one had an EUS-guided pseudocyst-duodenostomy stent insertion, and another had minimal access retroperitoneal pancreatic necrosectomy and a Roux-en-Y gastrojejunostomy. The nine patients with splenomegaly received pre- and per-operative platelet transfusions in order to try to maintain the platelet count. In addition, four patients had a splenectomy to control the platelet count: in one patient (the index case), this was a staged splenectomy, and in three others, it was performed synchronously at the beginning of the surgery.

The two initial Livocado procedures were especially complex but established the procedure. In the first patient (the index case) who was a non-drinker with massive splenomegaly, refractory thrombocytopenia, and cavernous transformation of the hepatic portal vein with multiple varices, it was impossible to continue the surgical procedure because of intra-operative plummeting platelet levels. A second operation with large-volume platelet transfusion could only go as far as releasing dense adhesions around the pancreas, spleen, and diaphragm as the patient had undergone a left-sided nephrectomy 5 years previously with extensive post-operative radiotherapy resulting in malrotation of the transverse colon with dense adhesions in the left upper quadrant of the abdominal cavity. An elective splenectomy was only possible on the third attempt following splenic vein embolization the day before, but pancreas resection was still not possible. The patient developed complications following release of dense adhesions related to transverse colon ischemia requiring an extended right hemicolectomy, end ileostomy, and mucous fistula. The patient went on to undergo a successful Livocado procedure and synchronous ileostomy reversal and was able to return work.

The second patient presented as an emergency and had required intensive care support with complicated chronic pancreatitis secondary to alcohol with portal and splenic vein thrombosis, intra-abdominal collections, splenic and peri-splenic abscesses, and sepsis, with a background of type 2 diabetes mellitus, COPD, and a previous EUS-guided duodeno-pseudocystostomy stent insertion. Following 1-month intensive care support and percutaneous drainage of the abscesses, a “limited” Livocado procedure was performed. Following pre-operative splenic artery embolization, a complex procedure was performed involving splenectomy, resection of splenic and peri-splenic abscesses, coring out of 75% of the pancreas from the tail towards the pancreatic head, a longitudinal pancreato-jejunostomy, and diaphragmatic repair with transversus abdominus flap. A completion of formal Livocado procedure was undertaken 17 months later following clinical stabilization.

All subsequent 16 procedures followed a more straightforward single-procedure surgical outcome. Overall the median (IQR) operative time was 6 h 37 min (5 h 17 min–7 h 10 min). The median (IQR) hospital length of stay was 13.5 (10–21.3) days. All patients had chronic pancreatitis on histopathology and 7 (39%) also had focal PanIN 1a or 1b lesions. Nine (50%) patients had post-operative complications, two with Clavien-Dindo grade I, 5 with grade II, and one each with grades III and IV. There was no peri-operative or 90-day mortality.

### Patient follow-up

Median (IQR) length of follow-up was 32.5 (21–45.75) months. Four patients were lost to follow-up after a median (IQR) of 25 (19.5–30.5) months, two patients who had moved abroad, and the other two had stopped attending clinic after 15 months and 29 months. Two patients died following hospital readmission, the first from a cardiovascular accident at 3 months, and the second from decompensated alcoholic liver disease and emphysematous cystitis with *Klebsiella pneumoniae* secondary sepsis at 19 months.

**Fig. 3:**
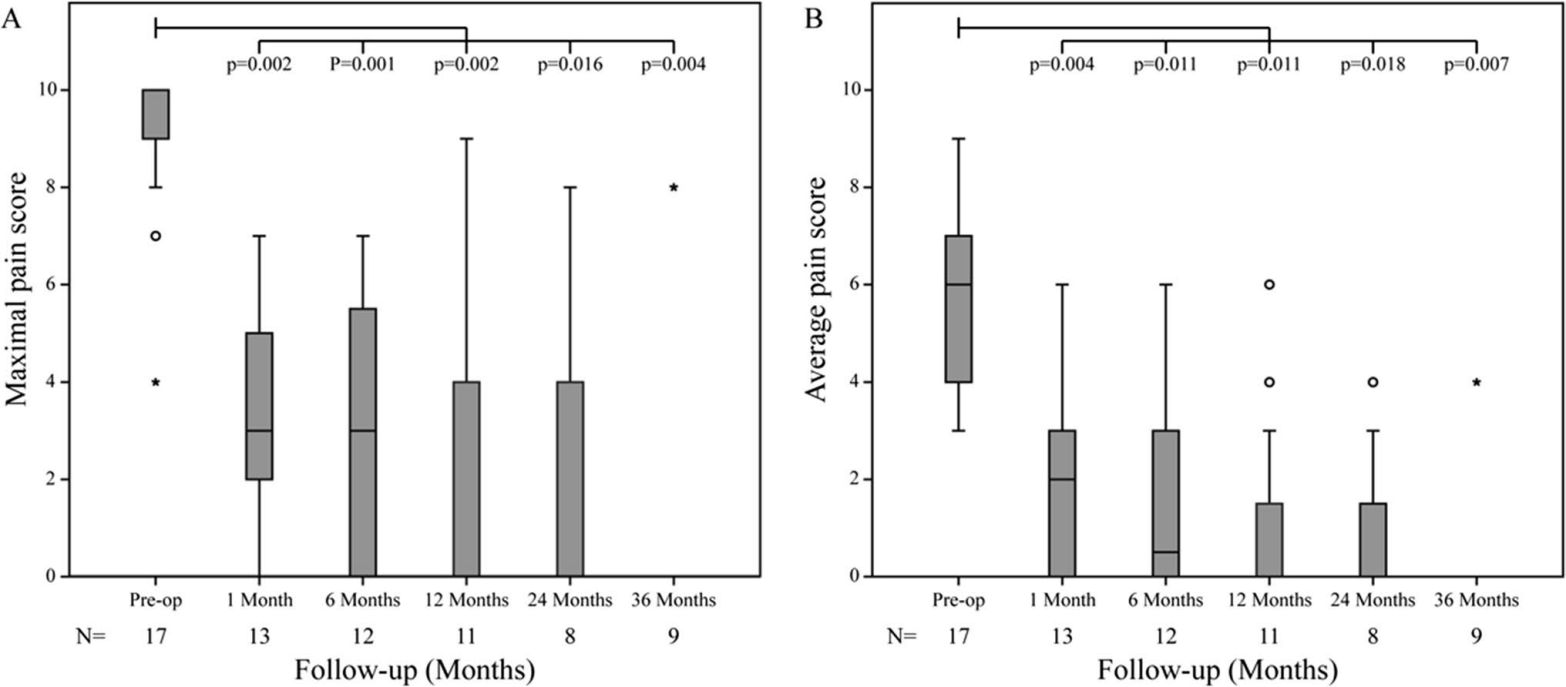
Pain scores pre-operatively and during follow-up. **a** Maximal reported pain scores. **b** Average reported pain score

**Fig. 4:**
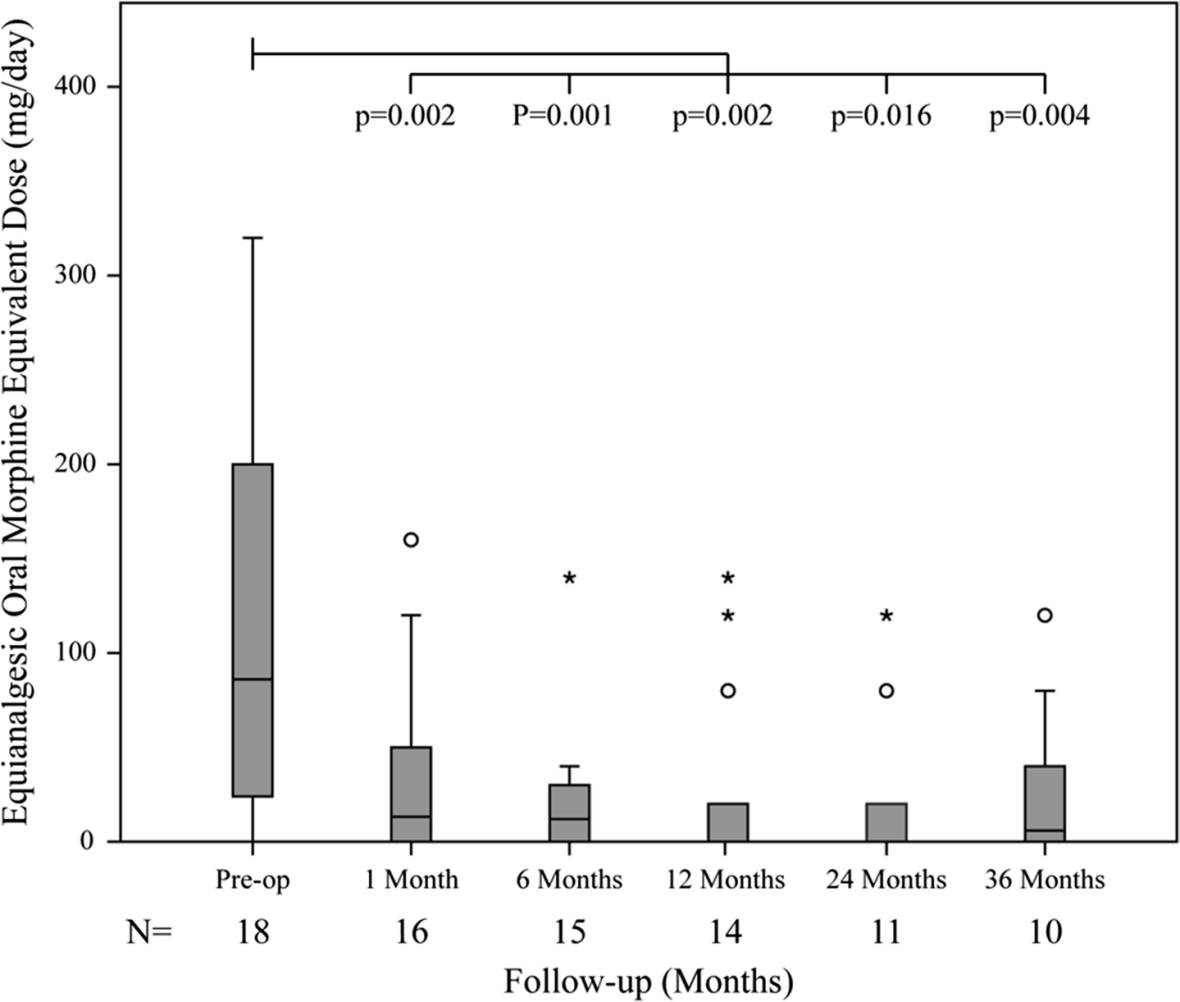
Equianalgesic oral morphine equivalent dose pre-operatively and during follow-up. Median, IQR, and range

Both maximal and average pain scores were significantly improved post-operatively; at 12 months, two-thirds of patients were completely pain free (Fig. 3a and b). Opiate analgesia use was also significantly reduced post-operatively (Fig. 4).

Postoperatively, 17 (94%) patients were diabetic, 14 requiring insulin, while three managed with oral anti-hyperglycemic medications (*p* = 0.041 compared to diabetes pre-operatively). The median (IQR) pancreatic exocrine replacement therapy lipase dose increased to 325,000 (242,500–450,000) lipase units/day post-operatively (*p* = 0.015 compared to pre-operative dosage). Pre-operatively, only six (33%) patients were employed, but post-operatively, 13 (72%) patients returned to employment (*p* = 0.01).

Three patients required re-intervention, one for revision of the jejunojejunostomy at 5-months, one was treated with IV antibiotics for acute cholangitis at 8 months, and a third required a biliary stent for a stricture at the ostium of the bile duct in the cored-out head of the pancreas.

## Discussion

**Fig. 5:**
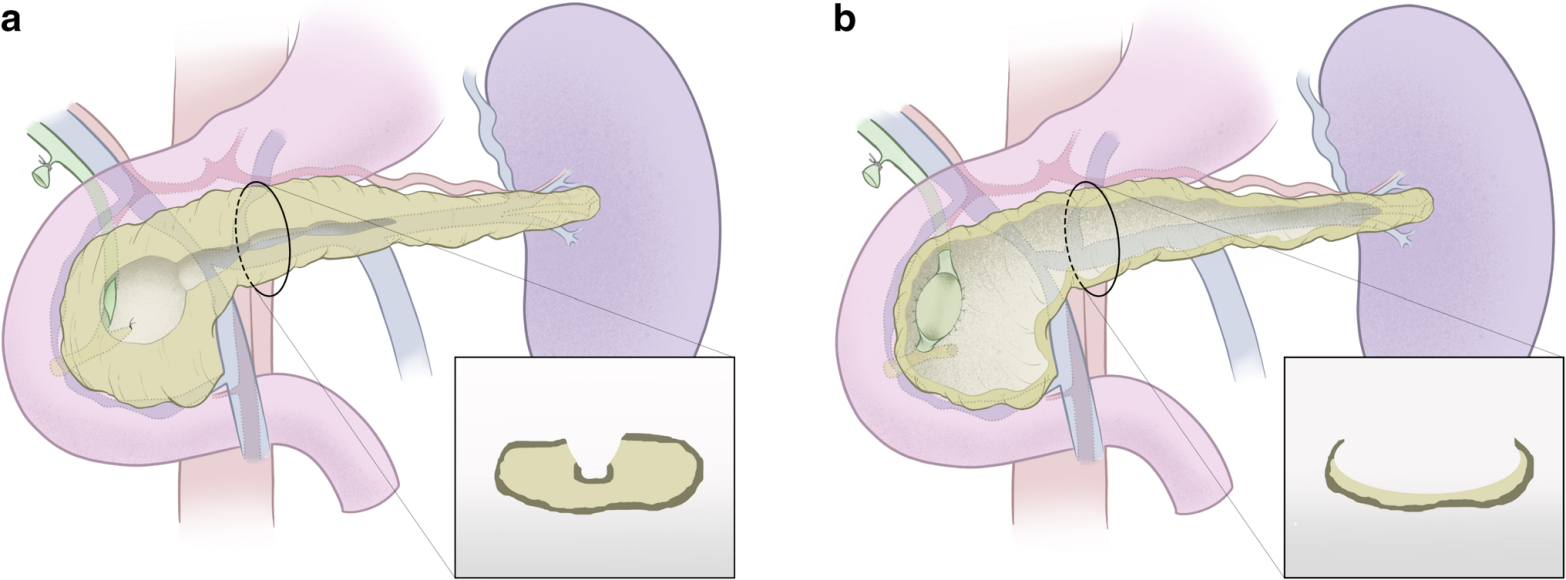
Whereas the duodenum-preserving pancreatic head resections such as the Frey procedure **a** aim to remove part of the head of the gland and improve drainage of the central and side branch pancreatic ducts, the Livocado procedure aims to remove all of the diseased parenchyma except for a rim of tissue posteriorly to avoid dissection into vascular planes and allow enough fibrous tissue around the rim for a secure Roux-en-Y pancreato-jejunostomy **b**

This novel operative approach was developed to be able to offer treatment to patients with end-stage chronic pancreatitis where conventional surgical options were at high risk or technically not feasible. Duodenum-preserving pancreatic head resections have been shown to be highly effective in improving symptoms [18–22, 33–35]. Whilst the duodenum-preserving pancreatic head resections such as the Beger, Berne, and Frey procedures target disease in the head of the pancreas with drainage of functioning tissue in the uncinate and body and tail of the pancreas, the Livocado procedure aims to remove all disease parenchyma including the head, uncinate process, body, and tail of the pancreas. The contrasting concepts between the Livocado and duodenum-preserving pancreatic head resections such as the Frey procedure are shown in Fig. 5a and b. In situations where a classical Kausch-Whipple partial pancreato-duodenectomy and the duodenum-preserving variants are both possible, the results in pain relief are comparable but the Beger-like procedures are superior in terms of post-operative complications and can be undertaken in more advanced cases [36]. The Hamburg group has described the Izbiki procedure with a “V”-shaped excision into the main pancreatic duct [37]. This was initially indicated for small duct disease as a drainage procedure, but was subsequently developed and combined with a duodenum-preserving pancreatic head resection [38]. As Izbicki et al. described “the Hamburg modification, which involved a V-shaped excision of the pancreatic body beyond the deep duodenum-preserving head resection, aiming to reach second-order and third-order pancreatic side branches. The concept behind this V-shaped excision was the idea of eliminating potential stenosis and prevention of stenosis that may appear as the disease, hypothetically resulting in better long- term outcomes [37, 38].” The indication is for patients with dominant head disease. In contrast, the Livocado procedure is for patients with involvement of the whole gland requiring a total pancreatotomy with removal of all diseased tissue but leaving a posterior capsule in patients at high risk because of vascular involvement.

The management of CP has followed a step-up approach, starting with lifestyle modification, medical therapy including analgesia, followed by interventional endoscopy, and finally, surgical resection is offered when all other measures have failed [2, 3, 18, 25]. There is now emerging data in support of improved outcomes in early (< 3 years after symptom onset) versus later surgery in more advanced disease stages in terms of long-term pain relief, reduced risk of pancreatic insufficiency, and reduced rates of re-intervention [25, 39]. However, the final options available are dependent on the extent of the disease, pancreatic exocrine function, presence of diabetes mellitus, and involvement of adjacent structures.

Vascular involvement is associated with major surgical risk, with splenic or portal vein thrombosis seen in 2.5–25% of all cases and in 10–37% of patients with alcohol-related CP, and is a relative contraindication to surgery [11–13]. The Livocado procedure was developed with the intention of removing as much diseased pancreas as possible without the necessity to enter into vascular planes or undertake vascular resections. The Hamburg modification of the duodenum-preserving pancreatic head resection resembles more of the Beger procedure when combined with a Puestow lateral pancreato-jejunostomy described originally in 1989 [38, 40]. The Hamburg modification aims to a perform “a longitudinal V-shaped excision of the ventral aspect of the body and the tail of the pancreas” in order to provide “sufficient drainage of the second-order and third-order pancreatic side branches” [40]. The Livocado procedure aims at a formal near-total pancreatectomy leaving only a rim of fibrous tissue for the pancreato-jejunostomy. The difference is reflected in the degree of post-operative diabetes mellitus of 94% in this study and 69% with the Hamburg modification [38]. In this Livocado series, all patients had vascular involvement, compared to 32 (6.45%) out 496 patients undergoing the Hamburg procedure reported in 2011 [12]. Series on surgery of patients with chronic pancreatitis and vascular involvement have reported increased operative time, operative bleeding, post-operative morbidity, and mortality [11, 12, 41, 42]. In this Livocado series, there was morbidity Clavien-Dindo grade ≥ III of 11% and no deaths compared to previous reports of 43.8–70.6% and 3–63%, respectively [11, 12, 41, 42].

The Livocado procedure resulted in significant improvements in pain score and daily opiate requirements. Both were significantly improved by first follow-up at 1 month and continued to improve at 6 months before reaching the best point at 12 months, when two-thirds of patients were pain-free. Patients undergoing the Livocado procedure compared with the previously described Liverpool duodenum-preserving and spleen-preserving near-total pancreatectomy (DPSPTP) were older (48.5 versus 40.8 years), had higher previous alcohol consumption (200 versus 140 units/day), smoked more (26 versus 20 pack-years), reported higher maximal pain scores (9 versus 8), and required higher daily opiate doses (86 versus 50 mg/day), respectively [24]. Patients in the Livocado group had greater vascular involvement (100% versus 27%) and peripancreatic organ involvement but with similar ASA grades [24]. Despite having higher pre-operative pain scores, higher opiate requirements, and more peripheral organ and vascular involvement, patients undergoing the Livocado procedure did equally as well as those who underwent DPSPTP in the later era (post-2003) in terms of length of stay (13.5 versus 13.5 days, respectively), peri-operative complications (Clavien-Dindo ≥ III) (11.1% versus 12.5%), and late complications (27.8% versus 33%), and also no 90-day mortality with either procedure [24]. Post-operative pain scores and opiate dose reductions were similarly significantly improved following both operations and most patients returned to work (72% versus 58%) [24]. These data compare well with that from published series of total pancreatectomy with reported mortality ranging from 2.9 to 20.6% and complication rates of 15.3 to 51.9% [36]. Although there is interest in total pancreatectomy with islet auto-transplantation in the treatment of chronic pancreatitis, this procedure is not suitable for adults with CP who have end-stage disease with exocrine and complete endocrine failure with few or no functioning islets present [43].

The indications and advantages between the Hamburg, Frey, and Livocado procedures can be contrasted as follows. The Hamburg group has reported on 500 consecutive patients having the Hamburg modification, the predominate operation in their series for chronic pancreatitis, and aims to preserve tissue with the aim of a functional improvement both exocrine and endocrine [38]. They Frey procedure is similar with 40–50% of the patients with chronic pancreatitis requiring surgery fulfilling the criteria for this procedure [21]. In contrast, the Livocado procedure is required in fewer than 5% of patients requiring surgery for end-stage chronic pancreatitis with vascular involvement. Thus, the advantage of the Livocado procedure is that without this procedure, no pancreatic surgery would have been undertaken in these patients because the surgical risks for a standard total pancreatectomy were too high and it would be pointless in removing only some of the diseased pancreatic tissue in terms of symptom control.

In conclusion, the in situ near-total pancreatectomy Livocado procedure enabled effective surgical treatment to a selective group of patients with complex end-stage chronic pancreatitis with debilitating pain who would otherwise be at high risk from conventional total pancreatectomy.
